# Co-infections in COVID-19 critically ill and antibiotic management: a prospective cohort analysis

**DOI:** 10.1186/s13054-020-03135-7

**Published:** 2020-07-09

**Authors:** Alexia Verroken, Anaïs Scohy, Ludovic Gérard, Xavier Wittebole, Christine Collienne, Pierre-François Laterre

**Affiliations:** 1 Department of Microbiology, Cliniques Universitaires Saint-Luc – Université Catholique de Louvain, Avenue Hippocrate 10, Brussels, Belgium; 2 Intensive Care Department, Cliniques Universitaires Saint-Luc – Université Catholique de Louvain, Avenue Hippocrate 10, Brussels, Belgium

International guidelines recommend the initiation of empirical antibiotherapy for possible associated bacterial pneumonia in COVID-19 critically ill yet further suggesting a rapid reassessment upon source documentation [1]. In this prospective cohort analysis, we investigated the respiratory co-infection rate in COVID-19 critically ill through the use of rapid molecular testing and measured its impact on antibiotic management.

This preliminary analysis was conducted over a 1-month period at the intensive care unit (ICU) of the Cliniques universitaires Saint-Luc and included all COVID-19 adult patients from whom a lower respiratory tract sample could be obtained. Specimens were conveyed to the microbiology laboratory where a FilmArray Pneumonia Panel *plus* test (FA-PNEU, BioFire Diagnostics, Salt Lake City, UT, USA) was performed. The FA-PNEU is an automated multiplex PCR test allowing direct detection of 15 bacteria with a semi-quantitative value, 3 atypical bacteria, 9 viruses, and 7 antimicrobial resistance genes within 1 h and 15 min [2]. FA-PNEU testing was done 24/7, and results were immediately called to the intensive care physician pursuing antimicrobial optimization.

Forty-one COVID-19 patients were admitted to ICU, and 32 could be included upon respiratory sample availability. The study population was comparable to previously described COVID-19 critically ill in terms of age, sex ratio, severity scores, comorbidities, and symptoms [3]. FA-PNEU was performed within a mean of 10 days following symptoms’ onset and a mean of 1 day following ICU admission. FA-PNEU results identified 13/32 (40.6%) patients with a bacterial co-infection as detailed in Table 1. *Staphylococcus aureus*, *Haemophilus influenza*, and *Moraxella catarrhalis* were the principal bacteria identified with significant genome copies. None of the 32 FA-PNEU tests identified atypical bacteria neither other respiratory viruses. Direct communication of FA-PNEU results led to speeded-up antibiotic modifications in 15/32 (46.9%) patients.

**Table 1:** FA-PNEU results and antibiotic management (this table should appear after the result section)

**Patient no.**	**Sample type**	**FA-PNEU results**	**Treatment switch initiated by FA-PNEU results**	
		**Detected pathogens (10**^**6**^ **≥ 10**^**7**^**)**	**Initial antibiotherapy**	**Subsequent antibiotherapy**
1	Sputum	*–*	None	None
2	ETA	*Pseudomonas aeruginosa*	None	Ceftazidime
3	ETA	*Moraxella catarrhalis*	None	Cefuroxime
4	ETA	*–*	None	None
5	ETA	*–*	None	None
6	Sputum	*–*	None	None
7	ETA	*–*	None	None
8	ETA	*–*	Cefuroxime	None
9	Sputum	*Haemophilus influenza*	None	Cefuroxime
10	ETA	*–*	None	None
11	ETA	*–*	Cefuroxime	None
12	ETA	*M. catarrhalis*	Cefuroxime	Cefuroxime
13	ETA	*Staphylococcus aureus*	None	Flucloxacilline
14	ETA	*–*	None	None
15	ETA	*–*	None	None
16	ETA	*Streptococcus agalactiae*	Cefuroxime	Cefuroxime
17	Sputum	*–*	None	None
18	ETA	*S. aureus*	None	Flucloxacilline
19	ETA	*–*	Cefuroxime	None
20	ETA	*–*	None	None
21	ETA	*S. aureus*	Amoxicilline - clavulanic acid	Amoxicilline - clavulanic acid
22	Sputum	*H. influenza*	None	Cefuroxime
23	ETA	*H. influenza*	None	Cefuroxime
24	ETA	–	Cefuroxime	None
25	ETA	*S. aureus*	Cefuroxime	Flucloxacilline
26	ETA	*–*	None	None
27	Sputum	*S. aureus + mecA/C*	None	Vancomycine
28	Sputum	*–*	None	None
29	Sputum	–	None	None
30	Sputum	–	Piperacilline-tazobactam	None
31	ETA	–	None	None
32	ETA	*S. aureus + mecA/C − H. influenza*	None	Vancomycine + cefuroxime

*ETA* endotracheal aspirate, *FA-PNEU* FilmArray Pneumonia Panel *plus* test

It is a known difficulty to adjudicate on the presence of a co-infection in COVID-19 patients particularly in critically ill. Clinical presentation, inflammatory markers, and bilateral radiological infiltrates lead to misperception and cannot be used in the diagnosis of a bacterial superinfection. As a consequence, empirical antibiotherapy is quasi-systematically initiated until microbiological documentation of co-infecting pathogens. Yet, current data on co-infections is limited. With the focus on intensive care settings, a case series in February 2020 analyzing 21 COVID-19 ICU patients reported no bacterial respiratory co-infections but 3 influenza infections [4]. A similar case series investigated in March 2020 stated none of the 15 COVID-19 critically ill had a bacterial co-infection neither were they tested positive for respiratory viruses [5]. No information however was available on how patients were tested neither on treatment strategy. In our setting applying generalized molecular screening for co-infection, the rate was 40.6% and the main detected pathogens were causal agents of community-acquired pneumonia.

As rapid molecular testing was performed within the shortest possible time following ICU admission, a majority of our patients did not receive empirical antibiotherapy while awaiting FA-PNEU result. Ultimately one third of the patients remained antibiotic-free over the entire process, and 5 patients had their antibiotics stopped following a negative FA-PNEU result. These antibiotic savings are crucial for COVID-19 critically ill known to have a long ICU stay with reported nosocomial infection rates as high as 31% [6].

To conclude, bacterial documentation is essential to assess co-infection in COVID-19 critically ill. The use of molecular diagnostic tools and the initiation of narrow-spectrum antibiotics are key elements of COVID-19 antimicrobial stewardship guidelines in critically ill. Studies on larger populations and in different geographical areas should be performed to outline analogous antibiotic saving strategies.

## Data Availability

The datasets used and analyzed during the current study are available from the corresponding author on reasonable request.

## Abbreviations

ICU: Intensive care unit
FA-PNEU: FilmArray Pneumonia Panel *plus* test

## References

[1] Poston JT, Patel BK, Davis AM. Management of critically ill adults with COVID-19. JAMA. 2020. https://doi.org/10:1001/jama.2020.4914. 10.1001/jama.2020.4914 32215647

[2] Weber DM, Wallace MA, Burnham CAD, Anderson NW. Evaluation of the BioFire FilmArray Pneumonia panel for detection of viral and bacterial pathogens in lower respiratory tract specimens in the setting of a tertiary care academic center. J Clin Microbiol. 2020. 10.1128/JCM.00343-20. 10.1128/JCM.00343-20 PMC7315030 32321782

[3] Yang X, Yu Y, Xu J, Shu H, Xia J, Liu H, Wu Y, Zhang L, Yu Z, Fang M, Yu T, Wang Y, Pan S, Zou X, Yuan S, Shang Y. Clinical course and outcomes of critically ill patients with SARS-CoV-2 pneumonia in Wuhan, China: a single-centered, retrospective, observational study. Lancet Respir Med. 2020. 10.1016/S2213-2600(20)30079-5. 10.1016/S2213-2600(20)30079-5 PMC7102538 32105632

[4] Arentz M, Yim E, Klaff L, Lokhandwala S, Riedo FX, Chong M, Lee M. Characteristics and outcomes of 21 critically ill patients with COVID-19 in Washington State. JAMA. 2020. 10.1001/jama.2020.4326. 10.1001/jama.2020.4326 PMC7082763 32191259

[5] Bhatraju PK, Ghassemieh BJ, Nichols M, Kim R, Jerome KR, Nalla AK, Greninger AL, Pipavath S, Wurfel MM, Evans L, Kritek PM, West TE, Luks A, Gerbino A, Dale CR, Goldman LD, O’Mahony S, Mikacenic C. Covid-19 in critically Ill patients in the Seattle Region - case series. N Engl J Med. 2020. 10.1056/NEJMoa2004500. 10.1056/NEJMoa2004500 PMC7143164 32227758

[6] Huang C, Wang Y, Li X, Ren L, Zhao J, Hu Y, Zhang L, Fan G, Xu J, Gu X, Cheng Z, Yu T, Xia J, Wei Y, Wu W, Xie X, Yin W, Li H, Liu M, Xiao Y, Gao H, Guo L, Xie J, Wang G, Jiang R, Gao Z, Jin Q, Wang J, Cao B. Clinical features of patients infected with 2019 novel coronavirus in Wuhan, China. Lancet. 2020; 10.1016/S0140-6736(20)30183-5. 10.1016/S0140-6736(20)30183-5 PMC7159299 31986264

